# Activation of FCGR2A enhances the antitumor efficacy of hPSC-derived CAR-M

**DOI:** 10.3389/fcell.2025.1698030

**Published:** 2026-01-12

**Authors:** Xinzhi Yang, Lu Li, Sijing Zhu, Shengtao Li, Xinlu Wang, Yuling Han, Liuliu Yang

**Affiliations:** 1 State Key Laboratory of Experimental Hematology, National Clinical Research Center for Blood Disease, Haihe Laboratory of Cell Ecosystem, Institute of Hematology and Blood Diseases Hospital, Chinese Academy of Medical Sciences and Peking Union Medical College, Tianjin, China; 2 Tianjin Institute of Health Science, Tianjin, China; 3 Key Laboratory of Organ Regeneration and Reconstruction, State Key Laboratory of Stem Cell and Reproductive Biology, Institute of Zoology, Chinese Academy of Sciences, Beijing, China; 4 Institute for Stem Cell and Regeneration, Chinese Academy of Sciences, Beijing, China; 5 Beijing Institute for Stem Cell and Regenerative Medicine, Beijing, China; 6 State Key Laboratory of Biomacromolecules, Institute of Biophysics, Chinese Academy of Sciences, Beijing, China

**Keywords:** chimeric antigen receptor macrophages (CAR-Ms), FCGR2A, human pluripotent stem cells(hPSCs), proinflammatory macrophage polarization, tumor immunotherapy

## Abstract

**Introduction:**

Chimeric antigen receptor macrophages (CAR‐Ms) represent a novel approach in cellular immunotherapy. Human pluripotent stem cells (hPSCs) provide an unlimited and renewable cell source, enabling scalable and standardized production of CAR‐Ms with consistent quality.

**Methods:**

In this study, we established a robust differentiation protocol to generate CAR‐Ms from hPSCs. To evaluate HER2‐directed hPSC‐derived CAR-M functionality, we first profiled HER2 expression across multiple tumor cell lines and identified SKOV3 as the optimal target due to its high HER2 level. CAR constructs incorporating intracellular domains from CD3ɛ, FCGR1A, FCGR2A, FCGR2B, and FCGR3A were introduced into hPSCs via lentiviral transduction.

**Results:**

Importantly, CAR expression did not impair hPSCs differentiation into macrophages. Functional assays revealed that all CAR-Ms exerted cytotoxic effects on HER2‐positive SKOV3 cells, with FCGR2A‐based CAR-Ms demonstrating the strongest activity. Furthermore, polarization of CAR‐Ms into a proinflammatory state significantly enhanced tumor‐killing efficacy, particularly in FCGR2A CAR‐Ms.

**Discussion:**

These findings highlight the potential of FCGR2A as an optimal signaling domain for CAR‐M design and underscore the therapeutic promise of proinflammatory polarized CAR‐Ms in solid tumor immunotherapy.

## 1 Introduction

Chimeric antigen receptor (CAR) T cell therapy has achieved significant clinical success in treating hematologic malignancies, with several products approved for clinical use (Brudno and Kochenderfer, 2024; Qi et al., 2022; Pasquini et al., 2020; Wang et al., 2023). However, its application in solid tumors remains limited due to multiple challenges (Albelda, 2024). The tumor microenvironment (TME) of solid tumors imposes both physical barriers and immunosuppressive signals that hinder CAR-T cell infiltration, persistence, and cytotoxic function (Maggi et al., 2024). Moreover, the heterogeneous and dynamic expression of tumor-associated antigens reduces the specificity and durability of CAR-T responses in solid tumor settings (Zhang et al., 2022). To overcome these limitations, CAR engineering has been expanded to innate immune cells, such as natural killer (NK) cells and macrophages (Tsahouridis et al., 2025; Morva et al., 2025; Liu Y. et al., 2024; Wang et al., 2024; Zhen, 2024). Among these, macrophages offer particular advantages owing to their intrinsic capacity to infiltrate solid tumors, perform phagocytosis, and present antigen to T cells. As abundant components of the TME, macrophages play a dual role in both innate and adaptive immunity (Chen et al., 2023; Chen et al., 2025; Liu et al., 2025; Lv et al., 2024). The development of engineered CAR-macrophages (CAR-Ms) is therefore emerging as a promising strategy to bypass the obstacles that limit CAR-T efficacy in solid tumors (Sloas et al., 2021; Liang et al., 2023; Liu Z. et al., 2024). Additionally, the use of human pluripotent stem cell (hPSC)-derived macrophages provides a renewable, standardized, and scalable cell source, enhancing the translational potential of CAR-M therapy (Cao et al., 2019; Park et al., 2024; Shen et al., 2024; Park et al., 2023; Zhang et al., 2020).

Structurally, CARs consist of an extracellular single-chain variable fragment (scFv) for antigen recognition, a transmembrane domain, and an intracellular signaling domain that initiates downstream immune activation (Liu Y. et al., 2024; Minguet et al., 2024; Kagoya et al., 2018; Wang et al., 2025). Early CAR-Ms studies commonly employed T cell–derived signaling domains such as CD28 or CD3ε to induce antigen-dependent phagocytosis, with signaling mechanisms analogous to Fc receptor–mediated antibody-dependent cellular phagocytosis (ADCP), and were primarily based on the human monocytic cell line THP-1 or the murine macrophage line RAW264.7 (Huo et al., 2023; Klichinsky et al., 2020; Zhang et al., 2019; Yang B. et al., 2024). While these CD28^−^CD3ε-based CAR-Ms have demonstrated tumor-specific phagocytosis and antitumor efficacy in preclinical models, the non-native nature of CD3ε in macrophages may constrain optimal activation and effector function (Zhang et al., 2020; Klichinsky et al., 2020). In addition, several studies have developed functionally specialized CAR-Ms to improve the tumor microenvironment. For instance, a HER2-targeted CAR incorporating the transmembrane and intracellular domains of CD147 upregulated matrix metalloproteinase (MMP) expression, thereby promoting extracellular matrix degradation and enhancing T cell infiltration (Zhang et al., 2019). Other studies compared intracellular signaling domains derived from TLR2, TLR4, TLR6, MerTK, and CD3ε, and found that CAR-Ms containing the MerTK signaling module exhibited stronger phagocytic and cytotoxic activities *in vitro* (Niu et al., 2021; Pan et al., 2022). Collectively, these findings highlight that most existing CAR-M designs rely on non-macrophage signaling modules and are based on immortalized cell lines rather than hPSC–derived macrophages, limiting their physiological relevance.

In contrast, macrophages naturally express a range of Fc gamma receptors (FcγRs), including the high-affinity FCGR1A (CD64), the activating intermediate-affinity receptors FCGR2A (CD32A) and FCGR3A (CD16A), and the inhibitory receptor FCGR2B (CD32B) (Bournazos et al., 2020). These receptors mediate ADCP and are essential for macrophage-mediated tumor clearance. Integrating FcγR-derived signaling domains into CAR constructs may better exploit macrophage-intrinsic signaling pathways and improve antitumor performance (Chen et al., 2025).

In this study, we utilized an hPSC-derived macrophage platform to engineer a panel of HER2-targeted CAR-Ms. All constructs shared a HER2-specific scFv (P1h3) and incorporated distinct intracellular signaling domains derived from CD3ε, FCGR1A, FCGR2A, FCGR2B, and FCGR3A. We systematically evaluated the differentiation potential, phenotypic characteristics, and antitumor activities of these CAR-Ms against the HER2-positive ovarian cancer line SKOV3. Furthermore, we confirmed that proinflammatory polarization could enhance their antitumor efficacy. Among all constructs tested, the FCGR2A-based CAR-Ms demonstrated the strongest cytotoxic activity *in vitro*, highlighting their potential utility for solid tumor immunotherapy.

## 2 Materials and methods

### 2.1 Differentiation of hPSCs into macrophages

An optimized differentiation protocol was employed to efficiently generate macrophages from hPSCs, specifically H9 and H1 cells (Cao et al., 2019; Cao et al., 2020). Cells were dissociated into small clusters using ReLeSR (STEMCELL Technologies) and seeded at a low density of 2 × 10^5^ cells per well onto Matrigel-coated 6-well plates. On day 1, cells were cultured in IF9S medium supplemented with 50 ng/mL BMP-4 (STEMCELL Technologies), 15 ng/mL Activin A (STEMCELL Technologies), and 1.5 μM CHIR99021 (Cayman Chemical). On day 3, the medium was replaced with IF9S containing 50 ng/mL VEGF, 50 ng/mL bFGF, 50 ng/mL SCF (R&D Systems), and 10 μM SB431542 (Cayman Chemical). On days 5 and 7, cells were maintained in IF9S medium supplemented with 50 ng/mL IL-6, 10 ng/mL IL-3, 50 ng/mL VEGF, 50 ng/mL bFGF, 50 ng/mL SCF, and 50 ng/mL TPO (all from R&D Systems). On day 9, cells were dissociated using TrypLE (Life Technologies) and resuspended in IF9S medium containing 50 ng/mL IL-6, 10 ng/mL IL-3, and 80 ng/mL M-CSF. On day 13, the medium was refreshed with the same cytokine composition. Monocytes were harvested on day 15 for downstream applications. To induce macrophage differentiation, monocytes were seeded onto FBS-coated plates and cultured in IF9S medium containing 80 ng/mL M-CSF. Proinflammatory macrophage polarization was induced by treatment with 100 ng/mL LPS (Sigma) and 20 ng/mL IFN-γ (STEMCELL Technologies), while anti-inflammatory macrophage polarization was induced by treatment with 20 ng/mL IL-4 (STEMCELL Technologies). All differentiation steps were performed under normoxic conditions at 37 °C and 5% CO_2_. IF9S medium was prepared as previously described (Cao et al., 2019; Cao et al., 2020). Briefly, IMDM and Ham’s F-12 nutrient mixture were mixed at equal volumes (117.25 mL each) and supplemented with PVA (5% stock, 50 μL; final 10 mg/mL), lipid mixture (100×, 250 μL; final 0.1%), ITS-X (100×, 5 mL; final 2%), α-monothioglycerol (1.3% stock, 750 μL; final 40 μL/L), ascorbic acid 2-phosphate (AA2P; 5 mg/mL stock, 3.2 mL; final 64 mg/L), GlutaMAX (200 mM, 2.5 mL; final 2 mM), non-essential amino acids (NEAA; 100×, 2.5 mL; final 1%), and penicillin–streptomycin (5000 U/mL, 1.25 mL; final 0.5%) to a final volume of 250 mL.

### 2.2 Flow cytometry

To evaluate the expression of CD14 and CD34 (both from BioLegend) in hematopoietic progenitor cells and monocytes, flow cytometry was performed. Cells were stained with fluorochrome-conjugated antibodies against CD14 or CD34, and isotype controls were included as negative controls. A blocking step was performed using Human TruStain FcX (Fc Receptor Blocking Solution, BioLegend, cat. no. 422301) to minimize non-specific and Fc receptor–mediated antibody binding. After staining, cells were washed with PBS and incubated at 4 °C in the dark for 30 min. Data were acquired using a FACSAria flow cytometer (BD Biosciences) and analyzed with FlowJo software.

To evaluate the CAR expression on macrophages, a two-step staining protocol was performed. Cells were first incubated with Human HER2/ERBB2 Protein His tag (Sino Biological) as the primary stain, followed by Human Anti-His Tag APC (R&D Systems) as the secondary stain. Subsequent staining and analysis procedures were performed as described above.

To assess HER2 expression in various tumor cell lines, cells were stained with an anti-HER2 antibody or isotype controls. Subsequent staining and analysis procedures were performed as described above.

To further characterize macrophage phenotypes, cells were stained with fluorochrome-conjugated antibodies against CD14, CD11B, CD80, CD206, and CD45 (all from BioLegend). Staining and analysis were conducted as described above.

### 2.3 Plasmid construction, lentiviral packaging, and transduction

CAR constructs were generated by standard molecular cloning and inserted into a third-generation lentiviral backbone (pCDH-CMV-MCS-EF1-Puro) with a puromycin selection region. Each construct consisted of a HER2-specific scFv, a transmembrane domain, and one of the intracellular signaling domains derived from CD3ε, FCGR1A, FCGR2A, FCGR2B, or FCGR3A. To label SKOV3 cells with mCherry, the mCherry coding sequence was cloned into an EF1a promoter-driven expression plasmid. All constructs were sequence-verified. Lentiviral particles were produced using the calcium phosphate precipitation method. HEK293T cells were seeded at a density of 5 × 10^6^ cells per 10-cm dish 1 day prior to transfection, reaching approximately 70%–80% confluency at the time of transfection. For each dish, the CAR transfer plasmid, the packaging plasmids psPAX2, and the envelope plasmid pMD2G were mixed at a mass ratio of 4:3:1 (typically 10 μg total DNA per dish; 5 μg transfer plasmid, 3.75 μg psPAX2, and 1.25 μg pMD2G). The DNA mixture was first combined with sterile water, followed by addition of 2 M CaCl_2_ (60 μL per dish). An equal volume of 2×HBS buffer (pH 7.05) was then added dropwise while vortexing gently. The mixture was incubated at room temperature for 5 min before being added dropwise to HEK293T cells. Supernatants were collected after 48–72 h, filtered through a 0.45 μm membrane, and concentrated. Target cells were transduced in the presence of 8 μg/mL polybrene. After 48 h, medium was refreshed. At 72 h post-infection, mCherry^+^ cells were sorted by flow cytometry or selected using 1 ug/mL puromycin for 48 h to obtain stable mCherry- or CAR-expressing populations.

### 2.4 Tumor cell lines and culture

BXPC-3, MCF7, MDA-MB-468, A549, PANC-1, SKOV3, and SKBR3 cell lines were obtained from ATCC. BXPC-3, A549, and PANC-1 cells were cultured in RPMI-1640 medium with 10% FBS and 1% penicillin–streptomycin (P/S). MCF7, MDA-MB-468, SKOV3, and SKBR3 cells were cultured in DMEM with 10% FBS and 1% P/S. All cell lines were maintained at 37 °C with 5% CO_2_.

### 2.5 RNA extraction, reverse transcription, and qPCR

Total RNA was extracted using TRIzol reagent (Invitrogen) according to the manufacturer’s instructions. RNA concentration and purity were assessed by NanoDrop spectrophotometry. Reverse transcription was performed using SuperScript IV (Invitrogen), and quantitative PCR (qPCR) was conducted using SYBR qPCR Master Mix (Vazyme) on a QuantStudio Real-Time PCR System (Applied Biosystems). Gene expression was calculated using the 2^−ΔΔCt^ method, with β-actin as the internal control.

### 2.6 RNA sequencing and analysis

Total RNA was extracted using TRIzol (Invitrogen) and DNase I treated using Directzol RNA Miniprep kit (Zymo Research) according to the manufacturer’s instructions. RNA-seq libraries of polyadenylated RNA were prepared using the TruSeq Stranded mRNA Library Prep Kit (Illumina) according to the manufacturer’s instructions. The libraries underwent sequencing with single-end 50-bp reads on the Illumina NovaSeq 6000 sequencer. Raw sequencing reads in BCL format were processed through bcl2fastq 2.20 (Illumina) for FASTQ conversion and demultiplexing. After trimming the adaptors with cutadapt v1.18, the sequencing reads were mapped to the human GRCh37 reference by STAR v2.5.2b (Dobin et al., 2013). Read counts per gene were extracted using HTSeq-count v0.11.2, and normalized through a regularized log transformation with the DESeq2 package v1.42.1 in R v4.3.3 for downstream analyses (Love et al., 2014; Anders et al., 2015).

Unstimulated macrophage dataset were obtained from our previously published study (Vandana et al., 2024), whereas an additional dataset containing anti-inflammatory macrophage samples was derived from this study. To integrate the two datasets, batch effects were corrected using ComBat implemented in sva v3.50.0, with dataset origin specified as the batch variable and group labels included in the model; the shared samples present in both datasets served as a bridge for harmonization (Leek et al., 2012). Principal component analysis (PCA) was performed before and after correction to assess whether batch effects were effectively removed. Differential expression analysis was performed using limma v3.58.1 on the batch-corrected expression matrix (Ritchie et al., 2015). Differentially expressed genes (DEGs) were defined as those with an absolute log_2_ fold change (|log_2_FC|) > 1 and a Benjamini–Hochberg adjusted P value (adj. P value) < 0.05. Volcano plots for DEGs between unstimulated macrophage and anti-inflammatory were generated using ggplot2 v3.5.1, and heatmaps of representative genes comparing unstimulated macrophage and anti-inflammatory were produced using pheatmap v1.0.12.

### 2.7 Immunofluorescence staining

Cells were fixed with 4% paraformaldehyde for 15 min, washed with PBS, and blocked with PBS containing 5% horse serum. Cells were incubated with primary antibodies against CD80, CD206 and CD68 overnight at 4 °C, followed by fluorescent secondary antibodies for 1 h at room temperature. Nuclei were counterstained with DAPI, and images were acquired using a fluorescence microscope. mCherry fluorescence was quantified using ImageJ software and “Mean” values were used for figure generation.

### 2.8 Statistical analysis

GraphPad Prism 9.0 software was used to conduct the statistical analyses and graph generation. N = 3 independent biological replicates were used for all experiments unless otherwise indicated. *P*-values were calculated by unpaired two-tailed Student’s t-test, multiple t-test or one-way ANOVA with a common control unless otherwise indicated. All data are presented as mean ± SEM. n.s indicates a non-significant difference. *P < 0.05, **P < 0.01 and ***P < 0.001.

## 3 Results

### 3.1 Generation and phenotypic characterization of hPSC-derived macrophages

**FIGURE 1:**
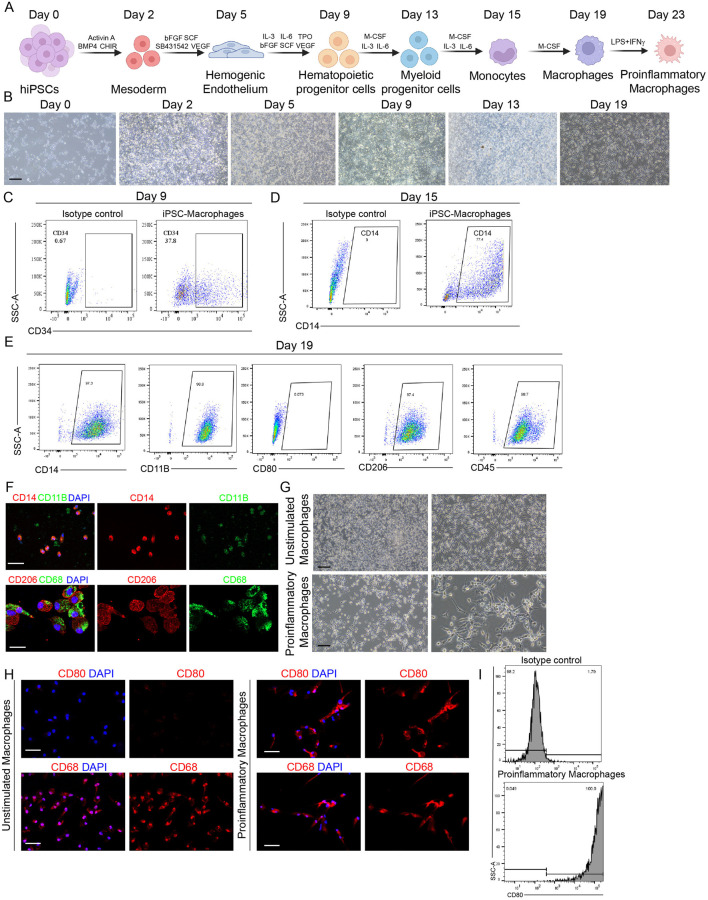
Generation and Phenotypic Characterization of hPSC-Derived Macrophages. **(A)** Schematic representation of the stepwise, serum-free differentiation protocol used to derive macrophages from human pluripotent stem cells (hPSCs). Schematic diagram also showed the polarization of unstimulated macrophages into proinflammatory macrophages using LPS (100 ng/mL) and IFN-γ (20 ng/mL). **(B)** Representative bright-field images showing morphological changes at defined stages of differentiation, including mesoderm, hemogenic endothelium, hematopoietic progenitors, and macrophages. Scale bar = 200 µm. **(C,D)** Flow cytometry analysis of CD34^+^ hematopoietic progenitors on day 9 and CD14^+^ monocyte-like cells on day 15. Antibody-omitted (blank) samples were used as negative controls to define gates and exclude nonspecific signals. **(E)** Flow cytometric characterization of mature macrophage surface markers CD14, CD11B, CD206, and CD45 on day 19. **(F)** Immunofluorescence staining of CD14 and CD11B in differentiated macrophages. Nuclei were counterstained with DAPI. Scale bar = 50 µm. **(G)** Bright-field microscopy images showing morphological changes before and after proinflammatory polarization. Scale bar = 200 µm. **(H)** Immunofluorescence staining of unstimulated and proinflammatory polarized macrophages showing co-expression of CD80 and CD68. Nuclei were counterstained with DAPI. Scale bar = 50 µm. **(I)** Flow cytometric analysis of CD80 expression. Isotype controls were used to define gating and exclude nonspecific signals. The schematic figures were created by using BioRender.com. BioRender export ID: CH296KA1AE.

To generate macrophages from hPSCs, we adopted a stepwise, serum-free differentiation protocol that mimics key stages of embryonic hematopoiesis, with modifications to improve efficiency and reproducibility. As illustrated in Figure 1A, the protocol involves sequential induction of mesoderm, hemogenic endothelium, hematopoietic progenitors, and finally, macrophage-lineage cells. Bright-field microscopy images captured at defined time points (Figure 1B) revealed distinct morphological changes associated with each stage of differentiation, including the transition from adherent, cobblestone-like progenitors to loosely attached, round monocyte cells and, eventually, macrophages with characteristic ruffled membranes. Flow cytometric analysis performed on day 9 demonstrated that the cell population expressed CD34, a well-established marker of hematopoietic progenitor cells (HPCs), indicating efficient commitment to the hematopoietic lineage (Figure 1C). By day 15, cells were positive for CD14, marking progression toward the monocyte lineage (Figure 1D). By day 19, the majority of cells exhibited high expression of mature macrophage markers, including CD14 (97.3%), CD11B (98.8%), CD206 (97.4%), and CD45 (98.7%), confirming terminal differentiation into macrophages (Figure 1E). The yield and purity of CD14^+^ monocytes achieved by our differentiation protocol were as follows: starting with 2 × 10^5^ cells, we obtained about 3.06 × 10^6^ cells by day 19, with a CD14^+^ purity around 97.3%, yielding over 14.89 CD14^+^ monocytes per initial cell. Notably, expression of the co-stimulatory molecule CD80 was negligible, suggesting that these cells resemble resting, unstimulated macrophages rather than activated, pro-inflammatory macrophages.

To further validate the identity of the differentiated cells, immunofluorescence staining was performed for key macrophage markers. Consistent with flow cytometry results, the cells strongly expressed CD14, CD11B, CD206, and CD68 (Figure 1F), demonstrating acquisition of a macrophage-specific phenotype. Together, these findings confirm that the differentiation protocol efficiently produces phenotypically mature hPSC-derived macrophages and that they are suitable for downstream functional assays, including CAR engineering.

To augment the antitumor potency of hPSC-derived CAR-Ms, we induced a proinflammatory phenotype in unstimulated CAR-Ms using lipopolysaccharide (LPS) and interferon-gamma (IFN-γ) stimulation (Figure 1A). Bright-field microscopy revealed marked morphological changes following polarization, with proinflammatory-polarized CAR-Ms exhibiting dendritic, spiky membrane protrusions—hallmarks of activated, proinflammatory macrophages (Figure 1G). Immunofluorescence analysis confirmed the co-expression of the proinflammatory macrophage surface marker CD80 and the pan-macrophage marker CD68 compared to unstimulated macrophages, further supporting successful polarization (Figure 1H). Flow cytometry analysis corroborated these findings by demonstrating a significant upregulation of CD80 expression in polarized cells compared to the unstimulated state (Figure 1I), validating the establishment of a proinflammatory-like phenotype.

To assess the plasticity of hPSC-derived macrophages and their ability to polarize into an anti-inflammatory phenotype, we further polarized these cells under M2-polarizing conditions (Supplementary Figure S1A). M2-polarized macrophages exhibited a rounded morphology under bright-field microscopy, distinct from the morphology observed under pro-inflammatory conditions (Supplementary Figure S1B). Immunofluorescence staining showed robust expression of CD68 and the M2-associated marker CD206 (Supplementary Figure S1C). Consistently, flow cytometry analysis revealed a high frequency of CD14^+^CD206^+^ cells, confirming the efficient polarization of cells toward an anti-inflammatory M2-like phenotype (Supplementary Figure S1D). While they share some similarities, these unstimulated macrophages are clearly distinct from fully polarized anti-inflammatory macrophages. This distinction is evident in our RNA-seq analysis comparing the two populations, where the volcano plot revealed numerous differentially expressed genes and heatmap showed highly expressed genes in anti-inflammatory macrophages (Supplementary Figures S1E, F). These data demonstrate that hPSC-derived macrophages retain functional plasticity and can be polarized toward either pro-inflammatory or anti-inflammatory states in response to appropriate stimuli.

### 3.2 HER2 Expression profiling in tumor cell lines and generation of fluorescently labeled SKOV3-mCherry cells

**FIGURE 2:**
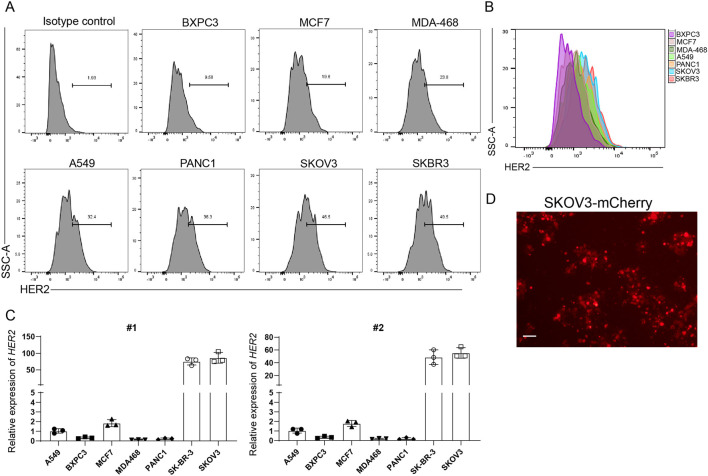
HER2 Expression Profiling in Tumor Cell Lines and Generation of Fluorescently Labeled SKOV3-mCherry Cells. **(A,B)** Flow cytometry analysis of surface HER2 protein expression in various tumor cell lines. Isotype controls were used to define gating and exclude nonspecific signals. **(C)** qRT-PCR analysis of HER2 transcript levels in tumor cell lines. β-actin served as the housekeeping gene for normalization. Each group included three independent biological replicates (N = 3). Data are presented as mean ± SEM. **(D)** Representative fluorescence microscopy image of SKOV3 cells stably expressing mCherry. Scale bar = 100 µm. The schematic figures were created by using BioRender.com. BioRender export ID: CH296KA1AE.

To select an appropriate target cell line for *in vitro* assessment of HER2-directed CAR-M cytotoxicity, we performed a comprehensive analysis of HER2 expression across a panel of human tumor cell lines. Flow cytometry analysis was performed to quantify surface HER2 protein levels, revealing that SKBR3 and SKOV3 cells exhibited high HER2 surface expression (Figures 2A,B). In contrast, other tested lines showed low or negligible HER2 levels, suggesting limited suitability for antigen-specific killing assays. To corroborate the flow cytometry data, we also conducted quantitative real-time PCR (qPCR) to evaluate HER2 transcript levels in the same panel of cell lines. Consistently, SKOV3 cells also demonstrated highest HER2 mRNA expression among all tested lines (Figure 2C), indicating active transcription and potential for robust protein translation over time.

To facilitate live-cell imaging and quantification during co-culture experiments, we established a fluorescent reporter cell line by stably transducing SKOV3 cells with a lentiviral vector encoding the fluorescent protein mCherry. The resulting SKOV3-mCherry cells exhibited stable, uniform fluorescence when observed under a fluorescence microscope (Figure 2D), allowing for real-time monitoring of tumor cell viability. This HER2-expressing, fluorescently labeled SKOV3-mCherry line thus serves as a robust and quantifiable *in vitro* model for functional evaluation of HER2-targeted CAR-M constructs.

### 3.3 CAR introduction does not affect hPSCs differentiation into macrophages

**FIGURE 3:**
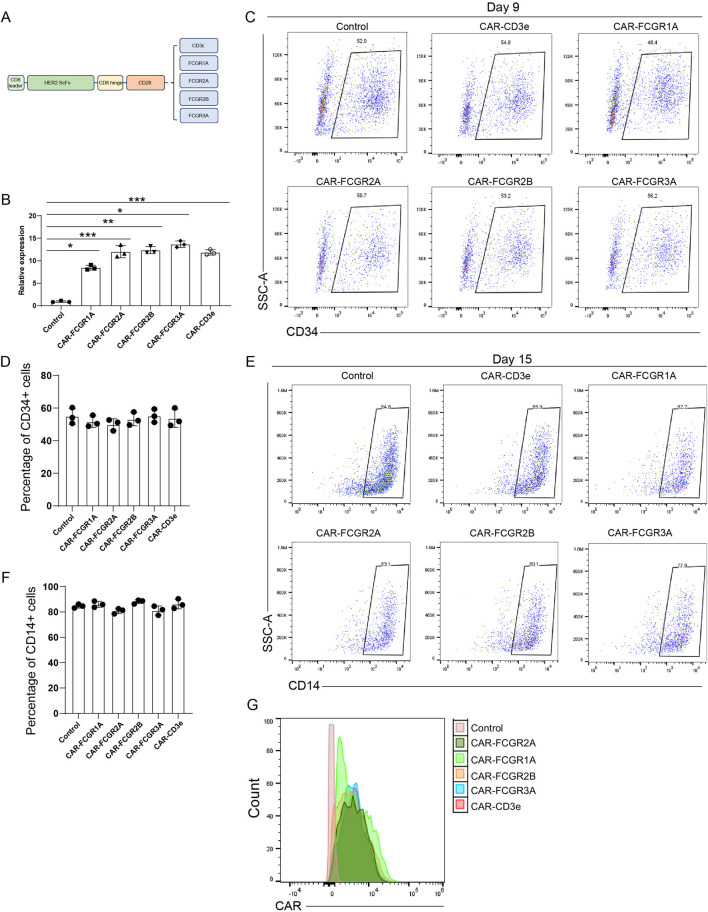
CAR Introduction Does Not Affect hPSC Differentiation into Macrophages. **(A)** Schematic representation of CAR constructs containing intracellular signaling domains derived from CD3ε, FCGR1A (CD64), FCGR2A (CD32A), FCGR2B (CD32B), and FCGR3A (CD16A). **(B)** qRT-PCR analysis of CAR expression in hPSCs prior to differentiation. β-actin served as the housekeeping gene for normalization. Each group included three independent biological replicates (N = 3). Data are presented as mean ± SEM. Statistical significance was assessed using two-way ANOVA, *P < 0.05, **P < 0.01, ***P < 0.001. **(C)** Flow cytometry analysis of CD34^+^ hematopoietic progenitor cells on day 9. Isotype controls were used to define gating and exclude nonspecific signals. **(D)** Quantification of CD34^+^ cell percentages shown in **(C)**. Data are presented as mean ± SEM from three independent biological replicates (N = 3). **(E)** Flow cytometry analysis of CD14^+^ monocyte cells on day 15. **(F)** Quantification of CD14^+^ cell percentages shown in **(E)**. Data are presented as mean ± SEM from three independent biological replicates (N = 3). **(G)** Flow cytometry analysis of CAR expression on the surface of hPSC-derived macrophages. The schematic figures were created by using BioRender.com. BioRender export ID: CH296KA1AE.

To determine whether the ectopic expression of CARs influences the differentiation trajectory of hPSCs into macrophages, we engineered a series of CAR constructs incorporating distinct intracellular signaling domains. Specifically, we designed five HER2-targeting CAR vectors, each incorporating a signaling module derived from CD3ε, FCGR1A, FCGR2A, FCGR2B, or FCGR3A (Figure 3A). These constructs shared an identical extracellular scFv targeting HER2 to ensure consistent antigen recognition, enabling direct comparison of downstream signaling effects.

Transduced hPSCs were subsequently subjected to a stepwise differentiation protocol optimized for macrophage generation. In parallel, we assessed CAR expression in undifferentiated hPSCs transduced with different CAR constructs (Figure 3B). To evaluate the impact of CAR expression on early hematopoietic commitment, we analyzed the expression of the hematopoietic progenitor marker CD34 on day 9 post-induction (Figure 3C). Flow cytometry revealed no statistically significant differences in the percentage of CD34^+^ cell populations among the CAR-expressing groups and the non-transduced control (Figure 3D), indicating that CAR integration does not disrupt mesodermal specification or early hematopoietic lineage development. Further phenotypic assessment was performed on day 15. All CAR-M groups exhibited comparable percentages of CD14^+^ cells to the control group (Figures 3E,F), suggesting that CAR expression does not impede the commitment to the myeloid lineage or affect macrophage differentiation efficiency. To further confirm that CAR expression on surface of macrophages at protein level, we performed flow cytometry. As shown in Figure 3G, different hPSCs-derived CAR-Ms displayed comparable levels of CAR expression, indicating that different intracellular signaling domains do not affect CAR presentation during macrophage maturation. Collectively, these findings demonstrate that the expression of CARs regardless of their intracellular signaling domain does not adversely affect the developmental trajectory of hPSCs toward macrophages.

### 3.4 Optimization of co-culture conditions and comparative evaluation of CAR-M cytotoxicity

**FIGURE 4:**
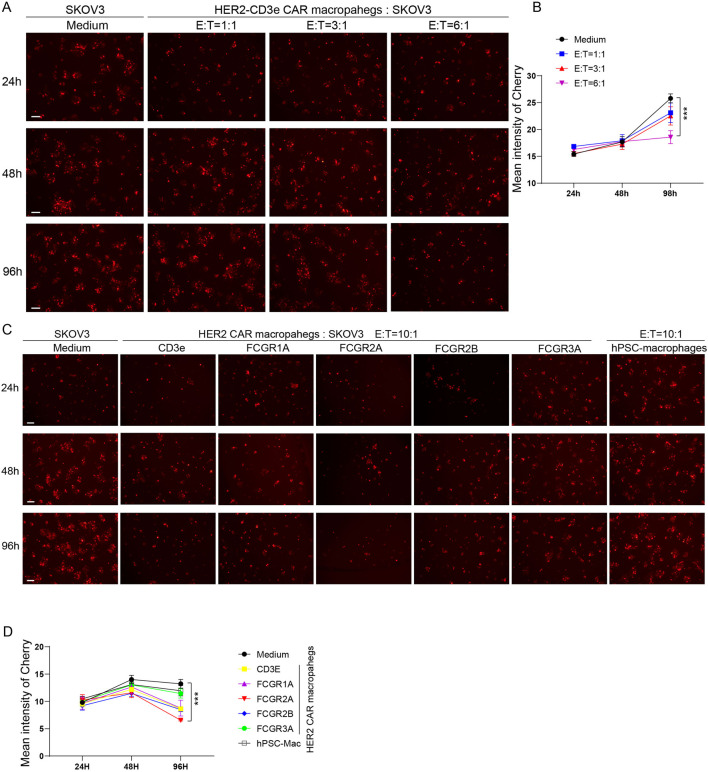
Optimization of Co-Culture Conditions and Comparative Evaluation of CAR-M Cytotoxicity. **(A)** Representative fluorescence microscopy images of HER2-CD3ε CAR-Ms co-cultured with SKOV3-mCherry cells at different effector-to-target (E:T) ratios (Medium, 1:1, 3:1, and 6:1), imaged at 24 h, 48 h, and 96 h. Scale bar = 100 µm. **(B)** Quantification of mCherry fluorescence intensity in SKOV3 cells at the indicated time points under different E: T ratios. Data are presented as mean ± SEM from three independent biological replicates (N = 3). Statistical significance was assessed using one-way ANOVA, with the Medium-only condition used as the control. ***P < 0.001. **(C)** Representative fluorescence microscopy images of SKOV3-mCherry cells at 24 h, 48 h, and 96 h after co-culture with CAR-Ms expressing different intracellular signaling domains (CD3ε, FCGR1A, FCGR2A, FCGR2B, and FCGR3A). Scale bar = 100 µm. **(D)** Quantification of mCherry fluorescence intensity shown in **(C)**. Data are presented as mean ± SEM from three independent biological replicates (N = 3). Statistical significance at 96 h was assessed using multiple t-test. The *p* values between CD3e v.s. FCGR2A = 0.0005; FCGR1A v.s. FCGR2A = 0.057; FCGR2B v.s. FCGR2A = 0.001; FCGR3A v.s. FCGR2A = 0.005; Medium v.s. FCGR2A = 0.005; hPSCs-Mac v.s. FCGR2A = 0.028; *P < 0.05, **P < 0.01, ***P < 0.001. The schematic figures were created by using BioRender.com. BioRender export ID: CH296KA1AE.

To establish optimal experimental parameters for assessing CAR-M-mediated cytotoxicity, we initially investigated the effect of varying effector-to-target (E:T) cell ratios using hPSC-derived HER2-specific CAR-Ms incorporating the CD3ε intracellular signaling domain. CAR-Ms were co-cultured with SKOV3-mCherry tumor cells at E:T ratios of 1:1, 3:1, and 6:1, and mCherry fluorescence intensity was monitored at 24 h, 48 h, and 96 h post co-culture (Figure 4A). A stop in the increase of fluorescence was detected in the 6:1 condition at 96 h (Figure 4B). These results suggest that extended co-culture duration and a higher E:T ratio are required for effective CAR-M–mediated tumor cell clearance. Consequently, the 10:1 E:T ratio at 96 h was adopted as the standard condition for all subsequent cytotoxicity assays.

Using this optimized setup, we next compared the antitumor efficacy of CAR-Ms engineered with different intracellular signaling domains: CD3ε, FCGR1A, FCGR2A, FCGR2B, and FCGR3A. Representative fluorescence microscopy images revealed that all CAR-M variants induced a reduction in SKOV3-mCherry signal at 24 h, 48 h, and 96 h, with the most pronounced effect observed at 96 h (Figure 4C). Quantitative fluorescence analysis confirmed a time-dependent increase in cytotoxicity across all constructs, with the CAR-Ms bearing the FCGR2A signaling domain exhibiting the most significant reduction in mCherry fluorescence intensity (Figure 4D). This superior antitumor activity is consistent with the known role of FCGR2A in mediating ADCP in native macrophages. Collectively, these findings highlight the potential of FCGR2A-based CAR-Ms as a highly effective platform for targeting HER2-positive solid tumor cells *in vitro*.

### 3.5 Construction of proinflammatory-polarized CAR-Ms and enhancement of antitumor activity

**FIGURE 5:**
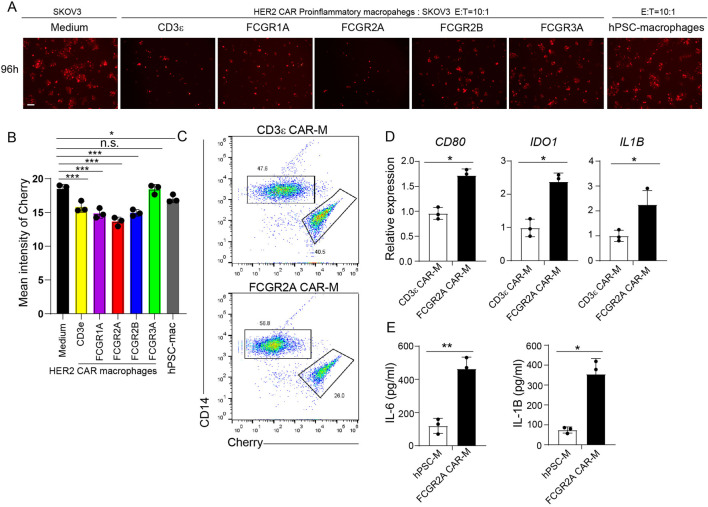
Construction of Proinflammatory-polarized CAR-Ms and Enhancement of Antitumor Activity. **(A)** Representative fluorescence microscopy images of proinflammatory polarized CAR-Ms co-cultured with SKOV3-mCherry cells at a 10:1 E: T ratio for 96 h. Scale bar = 100 µm. **(B)** Quantification of mCherry fluorescence intensity. Data are presented as mean ± SEM from three independent biological replicates (N = 3). Statistical significance was determined using t-test. The *p* values between Medium v.s. FCGR2A = 0.0009; CD3e v.s. FCGR2A = 0.02; FCGR1A v.s. FCGR2A = 0.09; FCGR2B v.s. FCGR2A = 0.05; FCGR3A v.s. FCGR2A = 0.001; hPSC-Mac v.s. FCGR2A = 0.01; *P < 0.05, **P < 0.01, ***P < 0.001. **(C)** Flow cytometry analysis of CD14 and mCherry in CD3ε-CAR-M and FCGR2A-CAR-M co-cultured with SKOV3 cells. **(D)** qRT-PCR analysis of proinflammatory associated genes (*CD80*, *IDO1*, *IL1B*) in sorted macrophages from CD3ε-CAR-M and FCGR2A-CAR-M groups after the killing assay. Data are presented as mean ± SEM from three independent biological replicates (N = 3). *P < 0.05. **(E)** ELISA assay was performed to analyze the proinflammatory cytokines (IL-6 and IL-1β) from CD3ε-CAR-M and FCGR2A-CAR-M groups after the killing assay. Data are presented as mean ± SEM from three independent biological replicates (N = 3). *P < 0.05. The schematic figures were created by using BioRender.com. BioRender export ID: CH296KA1AE.

To evaluate functional antitumor efficacy of proinflammatory-polarized CAR-Ms, we co-cultured them with SKOV3-mCherry ovarian cancer cells at an E:T ratio of 10:1 for 96 h (Figure 5A). Quantitative analysis of residual mCherry fluorescence revealed that most CAR-M variants induced substantial tumor cell killing, as evidenced by a marked decrease in fluorescence intensity. Among the constructs tested, CAR-Ms expressing FCGR2A also exhibited the most pronounced cytotoxic effect, leading to the greatest reduction in mCherry signal (Figure 5B). To further characterize the activation state of CAR-Ms following tumor cell engagement, we sorted macrophages from co-cultured cells and subjected them to qRT-PCR analysis. We found significantly higher expression of proinflammatory-associated genes (*CD80*, *IDO1*, *IL1B*) and proinflammatory cytokines in FCGR2A-CAR-M compared to CD3ε-CAR-M (Figures 5C–E). Collectively, these findings demonstrate that proinflammatory polarization enhances the tumoricidal activity of CAR-Ms, and that FCGR2A incorporation not only strengthens antitumor efficacy but also promotes a proinflammatory activation profile after target-cell killing.

## 4 Discussion

Chimeric antigen receptor macrophages (CAR-Ms) have recently emerged as a promising strategy in cellular immunotherapy, particularly for solid tumors where conventional CAR-T therapies face substantial limitations (Chen et al., 2025; Lei et al., 2024). Unlike T cells, macrophages possess inherent capabilities for tissue infiltration and stromal remodeling, enabling them to better navigate the complex architecture and immunosuppressive landscape of the tumor microenvironment (TME) (Pan et al., 2022; Anderson et al., 2021; Li et al., 2019; Cheng et al., 2020). In this study, we leveraged an hPSC-based differentiation system to generate HER2-specific CAR-Ms and systematically evaluated the impact of distinct intracellular signaling domains of different FcγRs on their differentiation potential and antitumor function. Our results demonstrate that the introduction of CAR constructs did not disrupt the differentiation of hPSCs into macrophages. Markers of hematopoietic and myeloid lineage commitment (CD34 and CD14, respectively) remained consistent across all constructs, indicating that CAR incorporation is compatible with macrophage development *in vitro*.

Building on current iPSC-based macrophage differentiation protocols (Cao et al., 2019; Cao et al., 2020), we established a renewable hPSC-derived CAR-M platform and used it to systematically compare intracellular signaling domains in a physiologically relevant context. In contrast to many previous CAR-M studies that relied on THP-1 or RAW264.7 cell lines (Huo et al., 2023; Klichinsky et al., 2020; Zhang et al., 2019; Yang B. et al., 2024), our use of hPSC-derived macrophages improves macrophage purity, reduces batch-to-batch variation, and more closely reflects primary human myeloid biology. This standardized platform therefore enhances both the translational potential and the reproducibility of CAR-M research.

Functional assays revealed that most CAR-M variants exhibited cytotoxic effects against HER2-positive SKOV3 cells. Notably, CAR-Ms harboring the *FCGR2A* signaling domain showed the most robust antitumor activity, particularly after polarization into the proinflammatory phenotype. These findings are consistent with the biological role of Fcγ receptors in macrophages. Different intracellular domains can transmit distinct categories of activation signals, particularly in FcγR-based CARs, where signaling is largely divided into activating versus inhibitory pathways. Activating FcγRs (including FCGR1A, FCGR2A, and FCGR3A) signal through the canonical ITAM–SYK–PI3K–PLCγ cascade, leading to Ca^2+^ flux, PKC activation, NF-κB/MAPK pathway activation, and pro-inflammatory cytokine production. In contrast, the inhibitory FcγR (FCGR2B) contains an ITIM motif that recruits SHIP1/SHP2 to counteract PI3K signaling (Bournazos et al., 2020; Bournazos et al., 2015). Therefore, functional differences among these intracellular domains primarily reflect variations in the magnitude and efficiency of this core activation program.

Previous studies have shown that FCGR2A knockdown suppresses M1 polarization and NF-κB phosphorylation while enhancing M2 polarization and STAT3 activation in intervertebral disc degeneration models (Luo et al., 2024). These findings suggest that FCGR2A may more effectively engage ITAM-dependent activation pathways, which could provide a plausible explanation for the superior killing efficacy observed in our FCGR2A-CAR-M. Moreover, the *FCGR2A* domain, when integrated into CAR constructs, enhance THP-1-derived macrophage phagocytosis and antitumor responses (Zhang et al., 2024). Our data align with this, highlighting *FCGR2A* as a superior signaling module for hPSC-derived CAR-M engineering.

The functional state of macrophages is highly influenced by their polarization. Proinflammatory macrophages are associated with tumoricidal properties, whereas anti-inflammatory macrophages are associated with immune suppression and tumor progression (Ghamangiz et al., 2025). Within the TME, macrophages are often skewed toward an anti-inflammatory phenotype, undermining therapeutic efficacy (Olsson et al., 2015; Ngambenjawong et al., 2017; Han et al., 2023). Our results showed that proinflammatory polarization, achieved via IFN-γ and LPS stimulation, significantly enhanced the cytotoxicity of hPSC-derived FCGR2A-based CAR-Ms.

Moreover, additional cytotoxicity readouts, phenotypic analysis of macrophages, and investigation of the *in vivo* efficacy of hPSC-CAR-Ms in solid tumor models are critical next steps for translating this technology. Maintaining a pro-inflammatory phenotype is essential for the success of CAR-M therapy *in vivo*. This challenge is precisely why our current research is focused on the foundational step of *in vitro* platform development and construct screening. In parallel, we are actively screening for key genetic factors that can enforce a stable pro-inflammatory state. The combination of these optimized hPSC-CAR-Ms with *in vivo* efficacy studies constitutes the focus of the future directions of CAR-M immunotherapy.

In addition to their role in immunotherapy, hPSC-derived macrophages offer a scalable and reproducible platform for disease modeling and cell-based interventions (Yang L. et al., 2024; Lang et al., 2018; Sun et al., 2022). Previous models using hPSC-derived macrophages have successfully recapitulated host-pathogen interactions and supported high-throughput drug screening, as demonstrated in *Mycobacterium* infection models (Han et al., 2019). Our study further supports the translational potential of hPSC-derived macrophages by validating their use in CAR-based engineering and functional enhancement through genetic and phenotypic modulation.

Collectively, our study identifies FCGR2A as a highly effective intracellular signaling domain for enhancing the antitumor activity of hPSC-derived CAR-Ms and establishes the critical role of proinflammatory polarization in amplifying their therapeutic efficacy. Combined with the engineering versatility and scalability of the hPSC platform, these findings provide a strong foundation for the development of next-generation CAR-M therapies tailored to overcome the unique challenges of solid tumor immunotherapy.

## Data Availability

The raw data supporting the conclusions of this article will be made available by the authors, without undue reservation.

## Supplementary material

Supplement Figure S1 Construction of Anti-inflammatory-polarized Macrophages. **(A)** Schematic diagram showing the polarization of unstimulated macrophages into anti-inflammatory macrophages using IL-4 (20 ng/mL). Created with BioRender.com (Export ID: CH296KA1AE). **(B)** Bright-field microscopy images showing morphological changes after anti-inflammatory polarization. Scale bar = 100 μm (left); Scale bar = 100 μm (right). **(C)** Immunofluorescence staining of anti-inflammatory polarized macrophages showing co-expression of CD206 and CD68. Nuclei were counterstained with DAPI. Scale bar = 50 μm. **(D)** Flow cytometry analysis of anti-inflammatory polarized macrophages showing co-expression of CD14 and CD206. **(E)** Volcano plot showed the differentially expressed genes in anti-inflammatory polarized macrophages versus unstimulated macrophages. **(F)** Heatmap showed the highly expressed genes in anti-inflammatory polarized macrophages versus unstimulated macrophages.
