# Supplementary figures and images for: Activation of FCGR2A enhances the antitumor efficacy of hPSC-derived CAR-M
Source: Front Cell Dev Biol. 2026 Jan 12;13:1698030. doi: 10.3389/fcell.2025.1698030 (PMC12833421; doi:10.3389/fcell.2025.1698030)

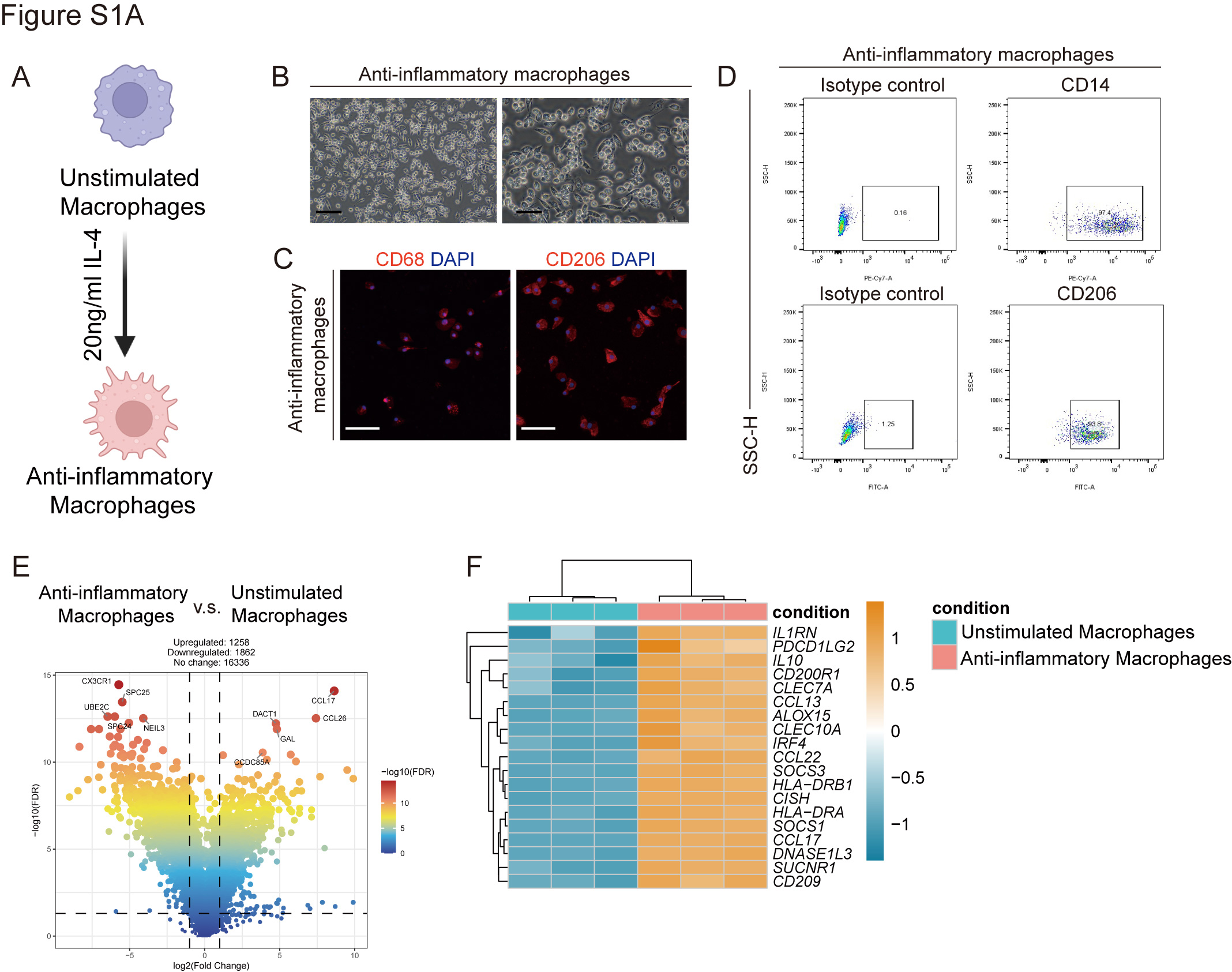

Supplement: Supplementary file 1 [file Image1.jpg]
